# Transient yellow discoloration of the nails for differential diagnosis with yellow nail syndrome

**DOI:** 10.1186/s13023-017-0711-4

**Published:** 2017-10-02

**Authors:** Anca Chiriac, Adrian Naznean, Cristian Podoleanu, Simona Stolnicu

**Affiliations:** 1 Department of Dermatology, Nicolina Medical Center, Iasi, Romania; 2 Department of Dermato-Physiology, Apollonia University Iasi, Iasi, Romania; 3 “P.Poni” Research Institute, Romanian Academy, București, Romania; 4 Department of Foreign Languages, University of Medicine and Pharmacy of Tirgu Mures, Tirgu Mures, Romania; 5 Department of Internal Medicine, University of Medicine and Pharmacy of Tirgu Mures, 38 Gheorghe Marinescu Street, 540139 Tirgu Mures, Romania; 6 Department of Pathology, University of Medicine and Pharmacy of Tirgu Mures, Tirgu Mures, Romania

**Keywords:** Yellow nail syndrome, Yellow discoloration, Nail

## Abstract

A differential diagnosis must be made between transient yellow discoloration of the nails and yellow nail syndrome. We highlight some practical aspects of yellow nail discoloration.

Sir,

We read with great interest the article by Vignes et al. recently published by the journal [1].

We would like to add some practical issues related to differential diagnosis of yellow nail discoloration.

A differential diagnosis must be made between transient yellow discoloration of the nails and yellow nail syndrome.

Yellow discoloration of the nails has been reported as an adverse reaction after some drugs such as: quinaqrine used for cutaneous lupus erythematosus [2], after topical use of 5-fluorouracil for the treatment of nail psoriasis (Fig. 1) [3], temsirolimus [4], or bucillamine for rheumatoid arthritis [5], retinoids [6] (Fig. 2). Furthermore, yellow discoloration of the nails has been described during hemodialysis [7], in patients diagnosed with non-Hodgkin lymphoma [8]. Yellow nails can be also observed in patients diagnosed with diabetes mellitus (Fig. 3a, b) [9], tobacco-associated use (Fig. 4), or after intense use of nail polish remover (Fig. 5). In recent years, skin adverse reactions induced by cetuximab have been lately reported; cetuximab is a monoclonal antibody against epidermal growth factor receptor (EGFR) used in the treatment of colorectal cancer [10]. Recently, yellowish distal discoloration was observed in our department in a 67-year-old female patient treated with cetuximab for colorectal cancer (Fig. 6).

**Fig. 1:**
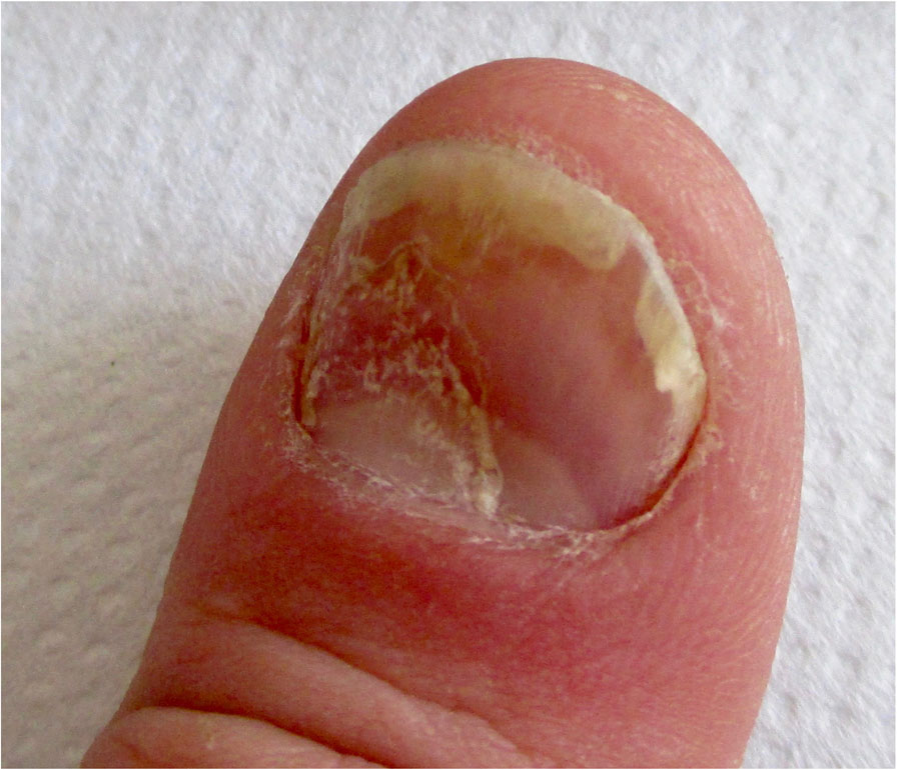
Diffuse *yellow* discoloration of the nail plate in a case of nail psoriasis treated with topical 5-fluorouracil (close view)

**Fig. 2:**
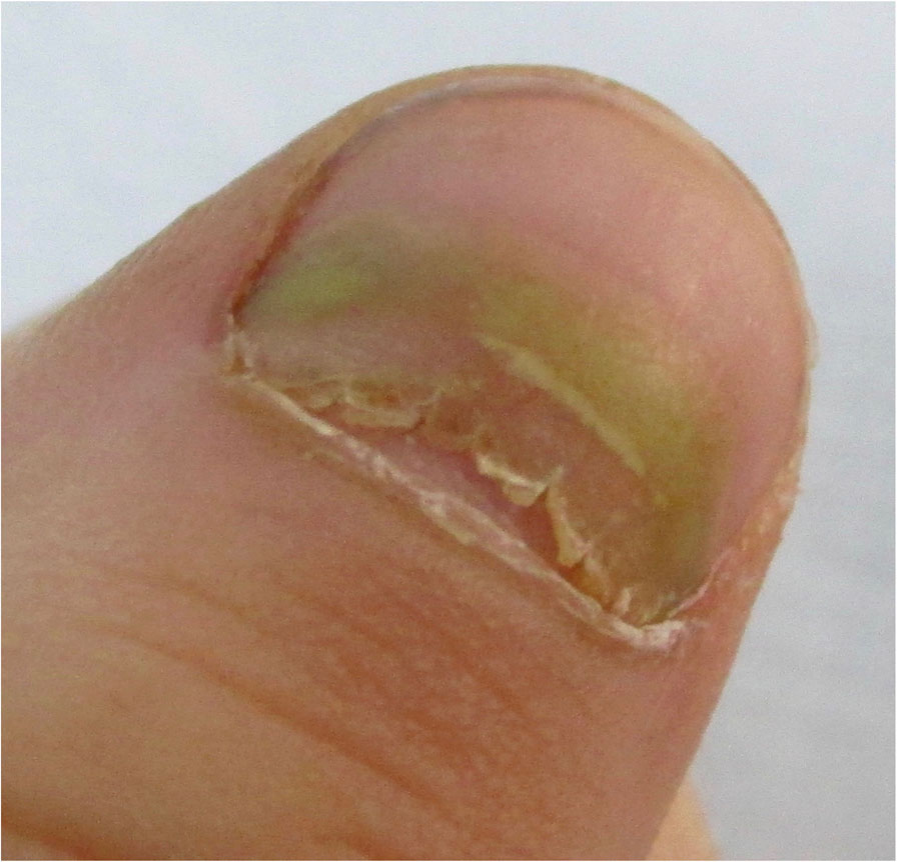
Yellowish discoloration of finger nails in a teenager while treated with systemic isotretinoin for acne

**Fig. 3:**
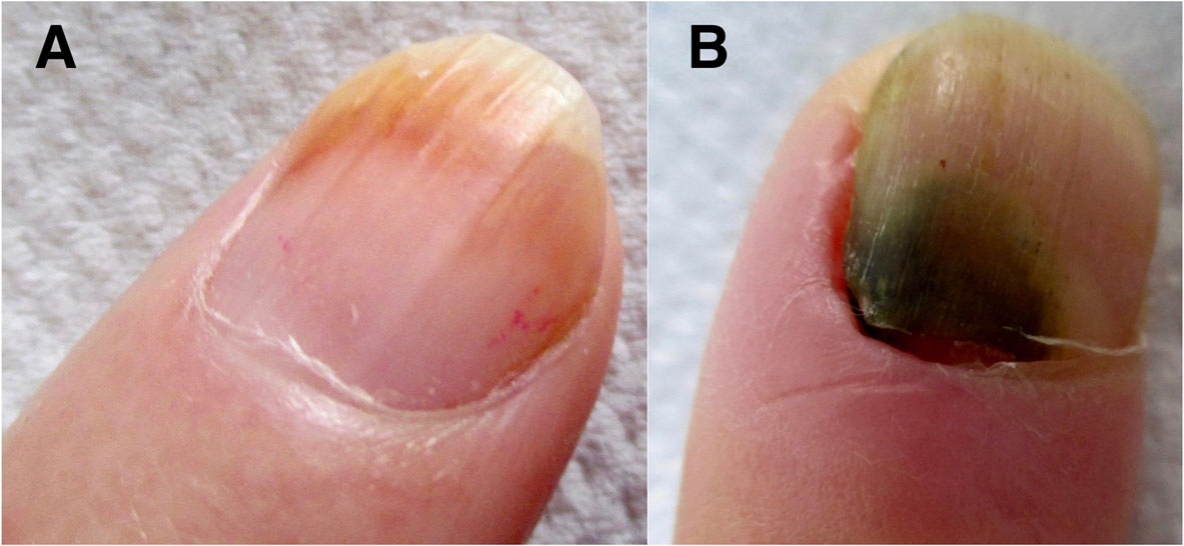
“Diabetic *yellow* nails” (**a**) and *yellow-green* discoloration of a nail in a diabetic patient due to *Pseudomonas aeruginosa* infection (**b**)

**Fig. 4:**
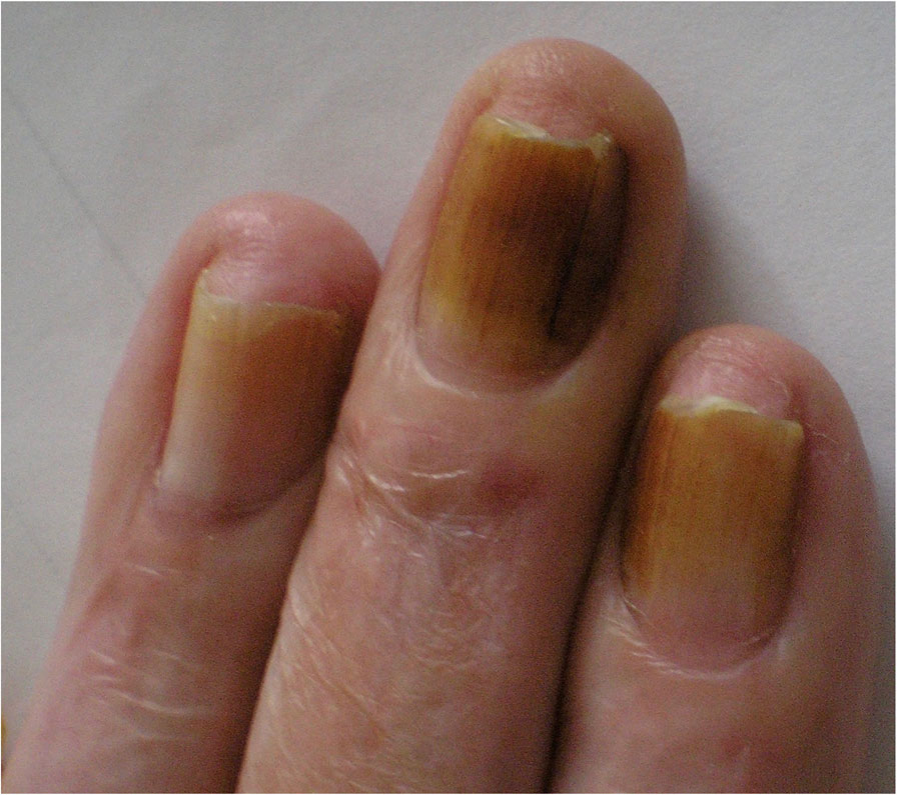
Tobacco associated *yellow* nails

**Fig. 5:**
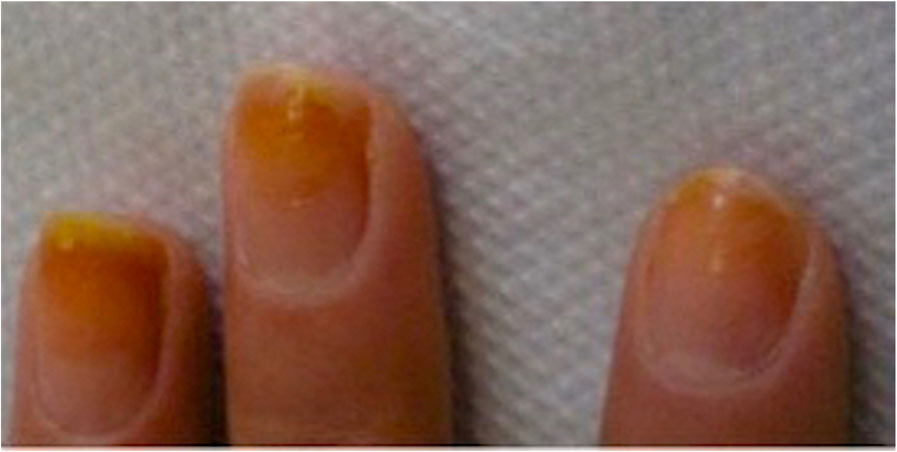
Transient yellow discoloration as a result of nail polish remover (acetone)

**Fig. 6:**
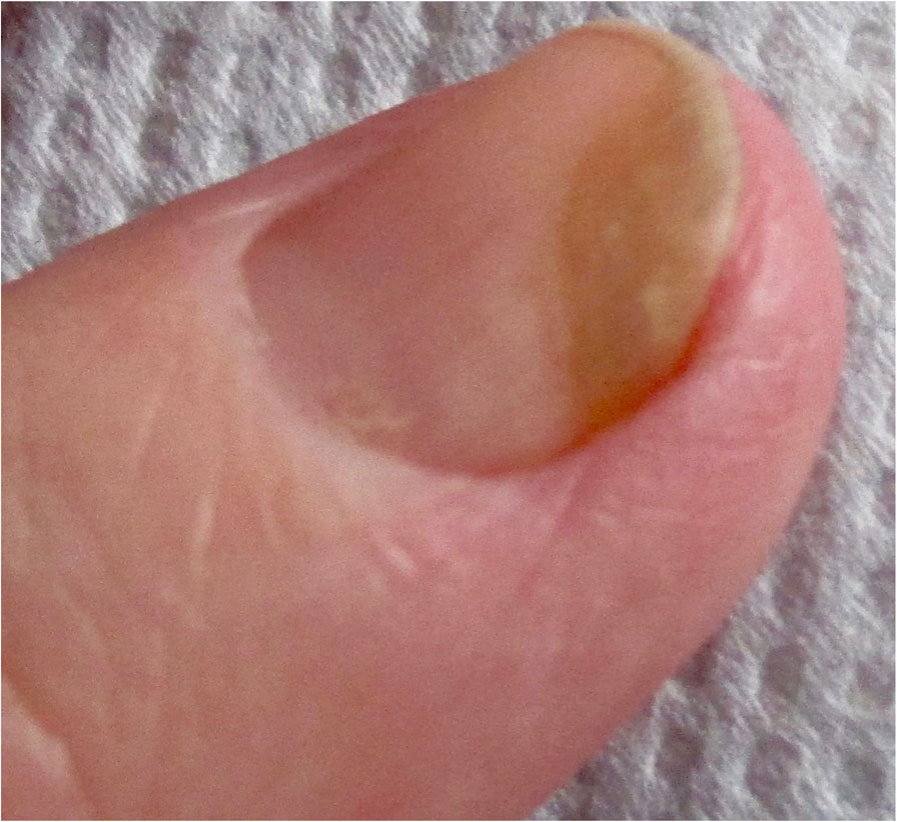
Distal *yellowish* discoloration of the nail during treatment with cetuximab
